# Genetics and Molecular Mapping of Black Rot Resistance Locus *Xca1bc* on Chromosome B-7 in Ethiopian Mustard (*Brassica carinata* A. Braun)

**DOI:** 10.1371/journal.pone.0152290

**Published:** 2016-03-29

**Authors:** Brij Bihari Sharma, Pritam Kalia, Devendra Kumar Yadava, Dinesh Singh, Tilak Raj Sharma

**Affiliations:** 1 Division of Vegetable Science, ICAR-Indian Agricultural Research Institute, New Delhi, India; 2 Division of Seed Science & Technology, ICAR-Indian Agricultural Research Institute, New Delhi, India; 3 Division of Plant Pathology, ICAR-Indian Agricultural Research Institute, New Delhi, India; 4 ICAR- National Research Centre on Plant Biotechnology, New Delhi, India; National Institute of Plant Genome Research, INDIA

## Abstract

Black rot caused by *Xanthomonas campestris* pv. *campestris* (Pam.) Dowson is the most destructive disease of cauliflower causing huge loss to the farmers throughout the world. Since there are limited sources of resistance to black rot in *B*. *oleracea* (C genome *Brassica*), exploration of A and B genomes of *Brassica* was planned as these were thought to be potential reservoirs of black rot resistance gene(s). In our search for new gene(s) for black rot resistance, F_2_ mapping population was developed in *Brassica carinata* (BBCC) by crossing NPC-17, a susceptible genotype with NPC-9, a resistant genotype. Out of 364 Intron length polymorphic markers and microsatellite primers used in this study, 41 distinguished the parental lines. However, resistant and susceptible bulks could be distinguished by three markers At1g70610, SSR Na14-G02 and At1g71865 which were used for genotyping of F_2_ mapping population. These markers were placed along the resistance gene, according to order, covering a distance of 36.30 cM. Intron length polymorphic markers At1g70610 and At1g71865 were found to be linked to black rot resistance locus (*Xca1bc*) at 6.2 and 12.8 cM distance, respectively. This is the first report of identification of markers linked to *Xca1bc* locus in *Brassica carinata* on B-7 linkage group. Intron length polymorphic markers provided a novel and attractive option for marker assisted selection due to high cross transferability and cost effectiveness for marker assisted alien gene introgression into cauliflower.

## Introduction

Black rot caused by the gram negative bacterium *Xanthomonas campestris* pv. *campestris* (*Xcc*) (Pammel) Dowson is one of the most destructive diseases of vegetable *Brassicas* wherever this crop is grown [1–2]. This disease has a wide geographical distribution [3] causing severe damage to the cauliflower crop resulting in 10–50% yield loss under congenial environmental conditions [4]. It causes a systemic infection in susceptible plants as pathogen enters leaves through the hydathodes at leaf margins or injuries. The disease spreads through vascular tissues, clogging vessels and producing V-shaped chlorotic lesions [5]. Managing this disease is very difficult as the bacterium spreads within and between fields by water splashes, wind, insects, machinery and irrigation. Therefore, resistance breeding would be rewarding and environmentally safe approach by exploring available genetic sources harbouring useful resistance genes effective against this devastating pathogen.

Most of the earlier black rot related studies had focused on *B*. *oleracea* (C genome) only which resulted in identification of limited sources of resistance [6–8]. Periodic outbreaks of the black rot disease have occurred worldwide, especially in the developing countries of Africa and Asia, where high temperatures and humidity favour the disease [9]. Although nine pathogenic races of *Xcc* have been identified [10], however race 1 and race 4 of these are predominant worldwide. The relative frequencies of their infection in *B*. *oleracea* group vary with the geographic regions [11]. There is no report yet showing availability of linked markers for *Xcc* resistance in *B*. *carinata*. However, there are some reports of mapping black rot resistance gene(s) in *B*. *napus* [11] and *B*. *rapa* [12–14]. Searching new gene(s) governing resistance to black rot from related monogenomic and digenomic species in ‘U’ triangle and pyramiding them into desirable backgrounds of *Brassica oleracea* would go a long way in combating this menace.

*Brassica carinata* A. Braun (BBCC, 2n = 34), an important oilseed crop originated in the Ethiopian plateau [15], is used as leafy vegetable in north east Africa. This has been reported to harbour genes controlling resistance/tolerance traits for biotic and abiotic stresses viz., resistance to black leg, black rot and tolerance to aluminium, salinity, heat and drought [11, 16–17]. The information about the genetic structure and molecular advancement in *B*. *carinata* specie is relatively meager [18]. Recently, a dense genetic linkage map in *B*. *carinata* covering a genetic distance of 2048 cM was constructed by Zou *et al*. [19] who also identified 136 blocks and islands conserved in Brassicaceae. Another linkage map of *B*. *carinata* was developed using a DH population derived from BcDH64/White- BcDH76 (YW) [18].

A variety of molecular marker techniques such as restriction fragment length polymorphism [20], random amplified polymorphic DNA [21–22], inter simple sequence repeat [23], microsatellite or simple sequence repeat [24] and amplified fragment length polymorphism [25] have been widely developed for gene tagging, diversity analysis and genetic linkage map construction in many crop species. The availability of large expressed sequence tag (ESTs) databases for many crop species provide a valuable resource for the development of molecular markers like intron length polymorphism (ILP). The variation in the intron sequences can be exploited as molecular markers [26]. Microsatellites or simple sequence repeats (SSRs) are one to six nucleotide motifs tandemly repeated up to 100 times at a locus. They are abundant in the genomes of higher organisms including human beings and plants.

Since there is no published report on linked markers for black rot resistance gene(s) and their gene specific location on the genome of *Brassica carinata*, the present investigation was undertaken to understanding genetics of black rot resistance in resistant genotype ‘NPC-9’, and identify intron length polymorphism (ILP) and microsatellite markers linked to resistance locus of *Xanthomonas campestris* pv. *campestris* (*Xcc*) race 1 in *Brassica carinata*.

## Materials and Methods

### Selection of parental material

The *Brassica carinata* accessions ‘NPC-17’ and ‘NPC-9’ were evaluated under laboratory as well as in field conditions against black rot pathogen *Xcc* race 1 consecutively during 2010 and 2011. The accession NPC-9 had minimum mean disease severity (0.10) and percent incidence (9%) in field conditions, while NPC-17 was observed with high disease severity (8.17) and percent incidence (96.66). Likewise, in laboratory conditions also the NPC-9 had minimum severity (0.25) and percent incidence (12.67), whereas NPC-17 showed maximum disease severity (7.66) and percent incidence (92), respectively. Based on the disease severity and incidence (%), the parental genotypes (NPC-17 and NPC-9) were selected for developing segregating progenies (F_2_, B_1_ and B_2_). The inbreds NPC-9 and NPC-17 were developed from the crosses viz., *B*. *carinata* early mutant × HC-2 and HC-2 × DLSC-1, respectively following pedigree method.

### Plant materials development

**Fig 1:**
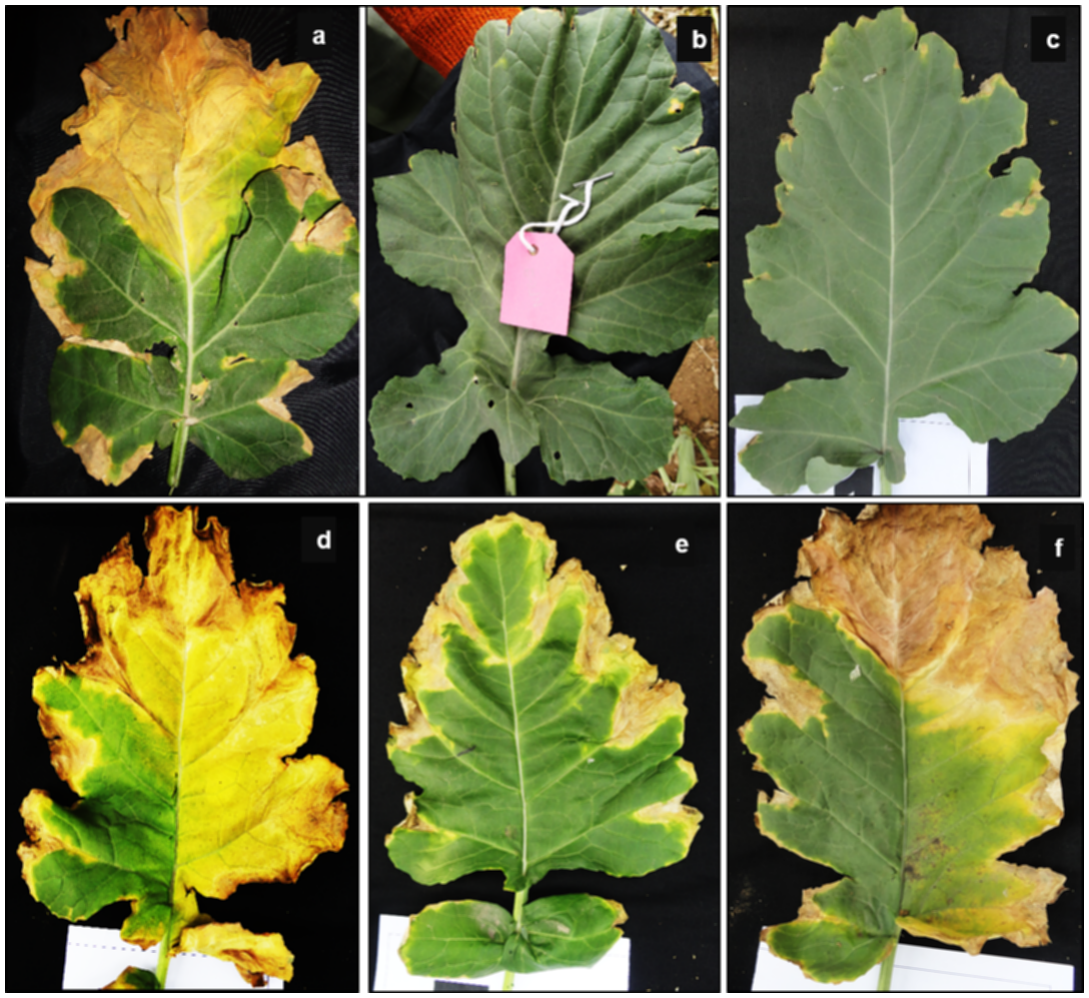
Phenotypic evaluation of resistant and susceptible parental genotypes and F_2_ population (NPC-17 × NPC-9) of *Brassica carinata* against *Xanthomonas* pv. *campestris* race 1. (a) Susceptible genotype ‘NPC-17’ (b) resistant genotype ‘NPC-9’, (c) leaves showing black rot disease resistance reaction in segregating F_2_ mapping population and (d-f) susceptible disease reaction in segregating F_2_ mapping population.

The accession NPC-17 (female) highly susceptible to black rot pathogen (Fig 1a) was crossed with resistant accession NPC-9 (male) (Fig 1b) to develop F_1_ hybrid. The F_1_ plants were selfed to generate F_2_ seed and pollinated simultaneously with NPC-17 (P_1_) and NPC-9 (P_2_) to generate backcross generations B_1_ and B_2_, respectively. All the segregating generations (F_2_, B_1_ and B_2_) were grown during October to March (2013–14) at the Research Farm of Division of Vegetable Science, ICAR-Indian Agricultural Research Institute, Pusa campus, New Delhi, India. All the plants in segregating generations (F_2_, B_1_ and B_2_) were precisely tagged and numbered after transplanting for their identity during phenotyping for disease and for drawing leaf samples for DNA extraction for genotyping. The F_2_ population was used for genotyping with putatively linked markers and three segregating generations, namely F_2_, B_1_, B_2_, were employed to study genetics of black rot resistance in *B*. *carinata*.

### Phenotyping of F_2_ mapping population

All the segregating generations (F_2_, B_1_ and B_2_) were screened against *Xcc* race 1 during November to December, 2013. The mean monthly weather data (S1 Table) for experimental period (October, 2013 –March, 2014) were collected from Division of Agriculture Physics, IARI, New Delhi. The bacterial strain (Accession number, ITCC-BH-0001; Delhi isolates, C1) of *Xcc* race 1 [27] was obtained from the Bacteriology Unit, Division of Plant Pathology, IARI, New Delhi, India and multiplied in yeast glucose chalk agar (YGCA) media at 25°C for 3 days. The culture was carefully scrapped from the media with sterilized slide. The scraped bacterial culture was mixed in 100 ml sterilized distilled water and mixed thoroughly by vortex and final concentration of 10^8^−10^9^ cfu/ml was made. The plants were first inoculated on 30^th^ day after sowing (DAS) by using leaf cut and dip technique [28]. The plants were inoculated by clipping the secondary veins at the margins with small scissor dipped in the bacterial suspension. The inoculation was carried out at 10 points per leaf on youngest leaves per plant in three replications. To maintain high humidity, sufficient moisture was maintained by frequent irrigation. Agrometeorological data (S1 Table) favouring disease establishment and development during phenotyping period November to December, 2013. The inoculated plants were assessed for disease reaction based on disease rating scale 0–9 and percentage of inoculated points in leaves showing symptoms were recorded as per scale given by Vicente *et al*. [11]. The disease reaction was recorded twice at 15 and 30 DAI and the final score of disease reaction at 30 DAI was used for grouping plants into resistant (Fig 1c) and susceptible (Fig 1d–1f) categories in segregating populations.

The total number of inoculated points and the number of points showing symptoms were recorded and the percentage of infected points were calculated as disease incidence. The severity of symptoms was assessed according to 0–9 scale based on the relative lesion size (0 = no symptoms; 1 = small necrosis or chlorosis surrounding the infection point; 3 = typical small V-shaped lesion with black veins; 5 = typical lesion half way to the middle vein; 7 = typical lesion progressing to the middle vein; and 9 = lesion reaching the middle vein). For segregation analysis plants were grouped on the basis of average disease severity and percent incidence into resistant and susceptible categories. Generally, plants with 0–5 disease severity and less than 50% disease incidence were categorized as resistant whereas those with 5–9 disease severity and more than 50% disease incidence as susceptible.

### Genomic DNA isolation and primer selection

Genomic DNA was isolated from the fresh young leaf tissues of both the parents and F_2_ plants using cetyl trimethyl ammonium bromide (CTAB) method [29]. The DNA was purified and quantified on 0.8% agarose gel by comparison with 50 ng/μl of standard uncut lambda (*λ*) DNA marker. DNA was diluted in sterile distilled water to a concentration of 25–30 ng μl^-1^. A total of 364 primers including 204 simple sequence repeats (SSR) (S2 Table) and 160 intron length polymorphic (ILP) markers (S3 Table) were used for polymorphic survey between the parents NPC-17 and NPC-9. Selected 125 SSR primer-pairs equally distributed to all linkage groups (LG C1–C9 and LG B1- B8) covering both the genomes of *Brassica carinata* [18]. Seventy nine SSR primer sequences were retrieved from *B*. *nigra* (BB) available publicly in the *Brassica* microsatellite information exchange (www.brassica.info/resource/markers/ssr-exchange.php). All the SSR primers were got synthesized from SBS Genetech Co. Ltd., China. One hundred sixty ILP primer sequences information used in this study were retrieved from *B*. *juncea* covering whole B genome (B1–B8) [30] and synthesized from Eurofins MWG Operon, Ebensburg (Germany).

### SSR and ILP marker analysis

Genotyping of mapping population was carried out with SSR markers by following PCR reactions in 0.2 ml thin walled sterilized PCR tubes. Amplifications were carried out in a total volume of 15 μl reaction containing 1.5 μl 10X PCR assay buffer with 17.5 mM MgCl_2_, 0.6 μl dNTP mix (10 Mm of each dATP, dTTP, dCTP and dGTP for direct use in PCR), 0.2 μl *Taq* DNA polymerase (5U μl^-1^), primer (10 μM) 1.5 μl each forward and reverse, 3 μl template DNA (25–30 ng) and sterile water (6.70 μl). The PCR chemicals used were from Himedia Laboratories, Mumbai, India. The PCR amplification reaction was carried out in a thermocycler (Eppendorf master cycler, Pro S model) with the initial PCR cycle at 94°C for 5 min for DNA denaturation followed by 35 cycles of 45s denaturation at 94°C, primer annealing at 50–57°C varying with primer-pairs for 1 min, 2 min extension at 72°C and final extension at 72°C for 7 min. The amplified products of SSRs were resolved by electrophoresis in 3% agarose gel (Hi media product, India) containing ethidium bromide in 1X TAE buffer (pH 8.0) at constant voltage (@ 5v/cm) for 3h. The size of amplified fragments was determined by co-electrophoresis of standard molecular weight marker. DNA profile was visualized on UV transilluminator and photographed by using Gel documentation System (Alpha Innotech, C-1000^™^). Intron length polymorphic marker analysis was performed by using initial denaturation at 94°C for 5 min, followed by 35 cycles of denaturation at 94°C for 1 min annealing at 53–62°C for 45s and elongation at 72°C for 3 min followed by a final extension at 72°C for 10 min. Amplified PCR products were analyzed on 2% w/v agarose gel. The remaining conditions set up was same as for SSR marker analysis.

### Bulk segregant analysis (BSA)

All the SSR and ILP primers were used for parental polymorphism to identify informative primers. To identify molecular markers putatively linked to black rot resistance locus, bulk segregant analysis [31] was performed. Equal quantity of DNA from ten resistant and ten susceptible F_2_ plants was mixed separately to create resistant and susceptible bulks. Polymorphic primers between parents and the resistant and susceptible bulks were tested on the genomic DNA of individual F_2_ plants employed in creating bulks. Putatively linked markers to the gene for resistance were examined by conducting SSR and ILP markers analysis of all 212 F_2_ plants.

### Statistical analysis

Disease reaction of each F_2_ plant to *Xcc* race 1 of the pathogen and data for marker were analysed by chi square (χ2) test [32] to determine goodness of fit to the expected segregation ratio of 3:1 (three resistant: one susceptible). A data matrix was generated and imported into MAPMAKER (3.0) [33]. The linkage between markers and resistance locus has been confirmed using this software. Markers within a group were ordered using the order command with LOD of 3.0. Map distances were calculated as centi Morgans (cM) using the Kosambi mapping function [34] and loci were ordered using the ‘sequence’ and ‘compare’ commands, with an LOD threshold score of 3.0.

## Results

### Genetics of black rot resistance

Inoculated susceptible plants started showing ‘V’ shaped symptom as light yellowish spot near the cut portion of leaf fifteen days after inoculation. All the plants in F_1_ generation as well as in F_2_, B_1_ and B_2_ generations were scored individually for black rot disease reaction. The plants were finally scored at 30 DAI for disease reaction and grouped into resistant and susceptible categories based on disease severity and incidence (%). All the F_1_ plants were resistant to black rot disease. This finding unveils dominant nature of resistance to black rot. Out of 212 F_2_ plants, 162 and 50 plants were found resistant and susceptible, respectively. On the comparison of observed segregation ratio with the expected in a χ2 test, 3:1 segregation ratio was observed with a χ2 value of 0.22 (P = 0.6–0.7) that fitted well with the expected ratio (Table 1). A total of 74 plants of B_1_ generation segregated as 40 resistant: 34 susceptible and fitted well with expected ratio (1:1). However, all the plants of B_2_ were found to be resistant. These findings advocated monogenic dominant control of resistance to black rot in *B*. *carinata* NPC-9.

**Table 1:** Genetic analysis of resistance to black rot in *Brassica carinata* populations derived from susceptible × resistant cross.

Genotype/populations	Disease reaction		Expected ratio	Calculated chi square value	*p* value
	Resistant^a^	Susceptible			
**NPC-17 (P**_**1**_**)**	0	10			
**NPC-9 (P**_**2**_**)**	10	0			
**F**_**1**_ **= NPC-17 X NPC-9**	30	0			
**B**_**1**_ **= F**_**1**_ **X P**_**1**_	40	34	1:1	0.48^b^	0.45–0.50
**B**_**2**_ **= F**_**1**_ **X P**_**2**_	48	0	1:0		
**F**_**2**_ **= F**_**1**_ **selfing**	162	50	3:1	0.22^b^	0.60–0.70

^a^ Resistant and susceptible refer to the disease reaction against *Xcc* race 1 on artificial inoculation. Critical value for the P = 0.05 significance level is 3.84.

^b^ Significant at given *p* value.

### Mapping of black rot resistance gene

#### Cross transferability and rapid identification of linked markers

**Fig 2:**
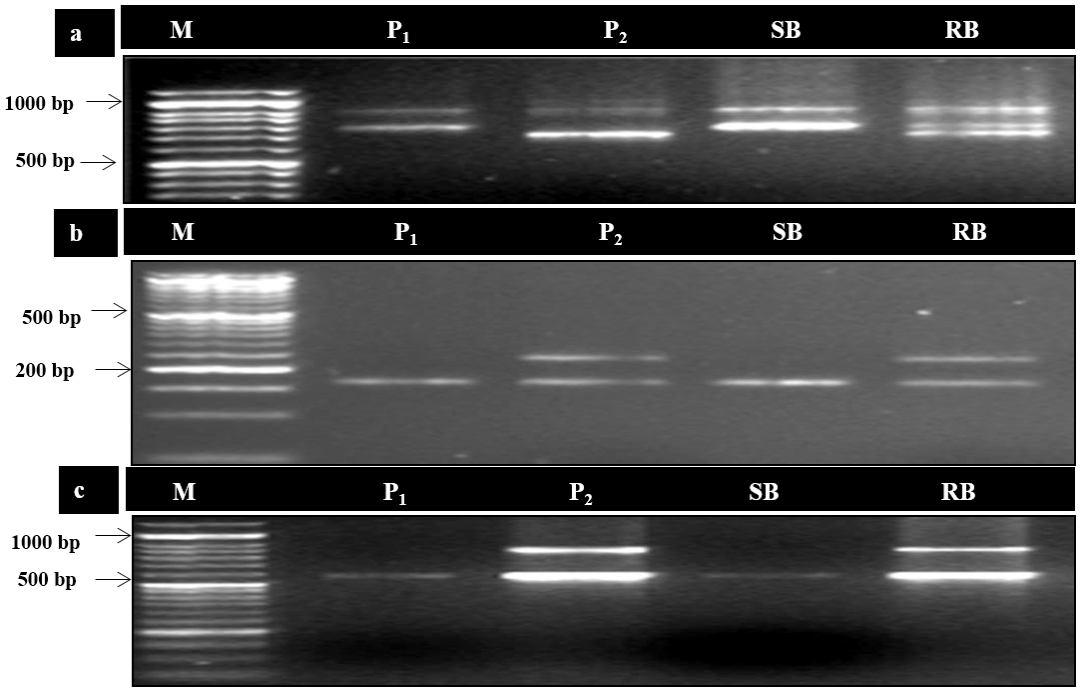
Bulk segregant analysis for the identification of linked markers. Markers able to differentiate resistant (RB) and susceptible bulks (SB) along with susceptible NPC-17 (P_1_) and resistant NPC-9 parents (P_2_) generating resistance specific band amplicon size approximate 700 bp of ILPAt1g70610 (a), 250 bp of SSR Na14-G02 (b) and 850 bp of ILP At1g7186 (c) markers.

Out of 364 (204 microsatellite and 160 ILP) primers used in parental polymorphic survey analysis, 41 distinguished the parental lines and, therefore, these were identified as polymorphic markers (S4 Table). Among these polymorphic markers, 23 were derived from *B*. *carinata* (B and C genome), 8 from *B*. *nigra* (B genome) and 10 from *B*. *juncea* (B genome). Two hundred and four microsatellite markers screened with two parental genotypes (NPC-17 and NPC-9) of *B*. *carinata* could exhibit 15.19% polymorphism level only. Intron length polymorphic primers (160) designed from *Brassica juncea* covering the entire B genome revealed 6.25% polymorphism in *B*. *carinata* ‘NPC-17’ and NPC-9 genotypes. These ILP markers of *B*. *juncea* exhibited high transferability percentage rates to the level of 93.75% in *B*. *carinata*. On testing polymorphic markers with contrasting resistant (R-bulk) and susceptible bulks (S-bulk), only three of them differentiated resistant and susceptible bulks generating resistant bulk-specific amplicon of approximately 700 bp (ILPAt1g70610), 250 bp (SSRNa14-G02) and 850 bp (ILPAt1g71865) as shown in Fig 2.

**Fig 3:**
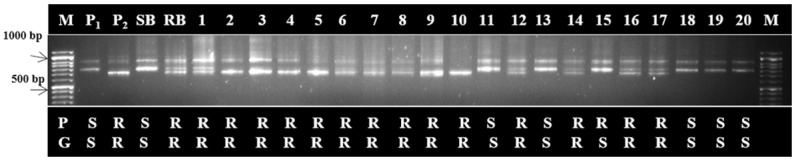
Genotyping pattern of F_2_ mapping population derived from the cross NPC-17 (P_1_) × NPC-9 (P_2_). A closely linked ILPAt1g70610 marker showing segregation for black rot resistance (R) and susceptibility (S). (P; phenotypic reaction, G; genotypic reaction, RB; resistant bulk and SB; susceptible bulk. M; 1Kb DNA ladder).

Single plant analysis with putatively linked markers (ILPAt1g70610, SSRNa14-G02, ILPAt1g7186) confirmed resistant and susceptible individuals. These linked markers to the gene resistant to *Xcc* were examined by conducting SSR and ILP markers analysis of all 212 F_2_ plants. Genotyping pattern of F_2_ individuals with the marker ILP At1g70610 closely linked to the gene of interest marker is given in Fig 3.

Observed segregation ratio of three molecular markers that fitted well with the expected ratio (Table 2) and the details of molecular markers linked to R gene conferring resistance to *Xcc* race 1 in *B*. *carinata* is given in Table 3.

**Table 2:** Segregation analysis of molecular markers with resistance locus (*Xca 1bc)*.

Marker	Observed F_2_ plants		Expected ratio	Calculated chi square value	*p* value
	Resistant^a^	Susceptible			
**Phenotype (*Xca1bc*)**	162	50	3:1	0.22^b^	0.50–0.70
**At1g71865 (ILP)**	161	51	3:1	0.11^b^	0.50–0.70
**At1g70610 (ILP)**	151	61	3:1	1.60^b^	0.10–0.50
**Na14-G02 (SSR)**	148	64	3:1	3.06^b^	0.05–0.10

^a^ Resistant and susceptible refer to the reaction of the 212 F_2_ plants derived from the cross of *Brassica carinata* (NPC-17 × NPC-9).

^b^ Significant at given *p* value.

**Table 3:** Molecular markers linked to R gene conferring resistance to *Xcc* race 1 in *B*. *carinata*.

Markers	ILP At1g71865	ILPAt1g70610	SSR Na14-G02
**Forward sequence (5`-3`)**	TTGCGTCTCCAGATCTCAA	TGGGTTATCTTCGCTGCGTT	TTCCCTTTATTGAGCAAGCTG
**Reverse sequence (5`-3`)**	GATCTTGCAGCTTGAATGAGTGA	GTCACCAACAGTTTGAGAGTCGA	TCCCGGTCGCTAAGATATTG
**Genetic distance from R locus (cM)**	12.8	6.2	30.1
**Motif type**	-	-	di GA/CT
**Repeat number**	-	-	17
**Annealing temperature (°C)**	53.1°C	54.9°C	55.0°C
**Size (bp)**	~ 850 bp	~ 700 bp	~ 250 bp
**Scoring pattern**	Dominant	Co- dominant	Dominant

#### Map construction and linkage group assignment

**Fig 4:**
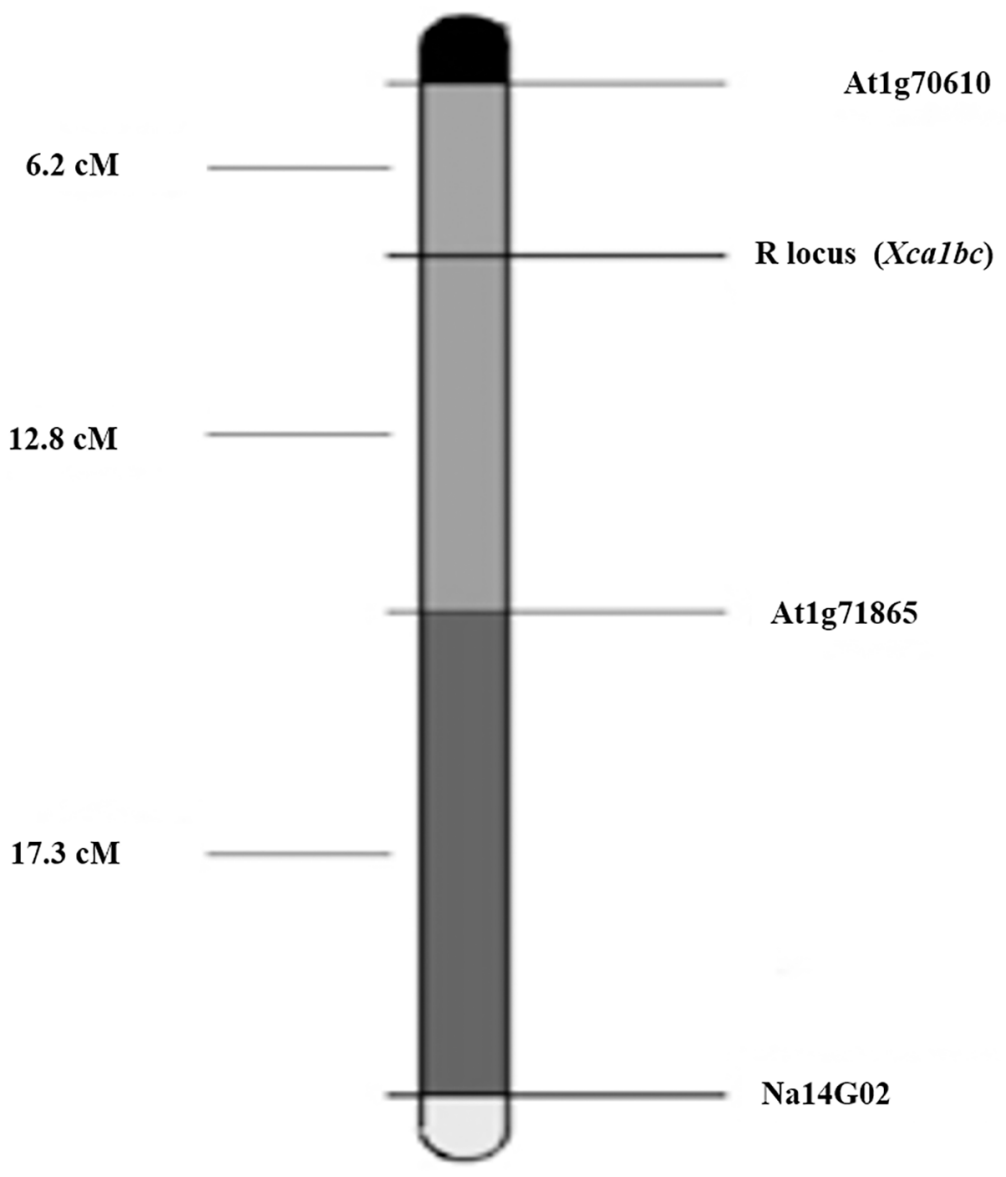
Map of R locus (*Xca1bc*) conferring resistance to *Xanthomonas campestris* pv. *campestris Xcc* race 1. Marker and R locus (*Xca1bc*) are depicted on the right side of the estimated map and the distances are on the left. This linkage group corresponds to LG 7 of B genome of *Brassica carinata*.

The linkage between markers and resistance gene (R) was confirmed using MAPMAKER (3.0) with LOD score 3.0. A linkage map of three markers along with the resistance locus covering 36.30 cM distance was developed (Fig 4).

Intron length polymorphic markers At1g70610 and At1g71865 flanked the resistant gene (R) at a genetic distance of 6.2 and 12.8 cM, respectively. However, SSR (Na14-G02) marker was far away from the resistant locus with a distance of 30.1 cM. This is the first report of identification of markers linked to black rot resistance locus (*Xca1bc*) in *Brassica carinata*. The chromosomal locations of the ILP (At1g70610 and At1g71865) and SSR (Na14-G02) markers linked to resistance gene were inferred from their position on the high-density genetic linkage map of *B*. *carinata* [18] and *B*. *juncea* [30], whereas SSR Na14-G02 marker was located at B-7 linkage group in conserved block E of *B*. *carinata* and ILP At1g70610 and At1g71865 markers were located in conserved block E of *B*. *juncea*.

## Discussion

### Genetics of black rot resistance

Exploring the new resistance sources from A and B genomes of alien *Brassica* species is one of the current priority areas for black rot resistance breeding in cauliflower. The accessions ‘NPC-9’ and ‘NPC-17’ of *B*. *carinata* were found as resistant and susceptible, respectively. The accession NPC-9 could be used for transferring resistance against *Xcc* race 1 into commercial susceptible varieties of *Brassica oleracea* group. Segregation analysis for *Xcc* resistance gene in three populations (F_2_, B_1_, B_2_) fitted well in expecting ratios indicated involvement of single dominant locus controlling resistance to *Xcc* race 1 in *B*. *carinata* accession NPC9. A pure Delhi isolate of *Xcc* race 1 was used for artificial inoculation, which facilitated the analysis of inheritance of resistance locus in more simplified and precise manner. On the basis of postulated gene-for-gene model, single dominant gene (R1) present in *B*. *carinata* revealed that resistance (incompatible interaction) with most of the pathogenic races differed from single dominant gene of *B*. *oleracea* (R3) for black rot resistance [2]. The co-segregation of disease reaction in individual F_2_ plants with putatively linked markers viz., ILP (At1g70610, At1g71865) and SSR (Na14-G02) showed good fit to 3: 1 ratio, confirming their linkage with black rot resistance locus (*Xca1bc*). On the basis of phenotypic and molecular marker segregation, we reached at a conclusion that R gene controlling the *Xcc* race 1 resistance was inherited monogenically with dominant nature in an accession ‘NPC-9’ of *B*. *carinata*. Therefore, it is important and feasible to transfer this black rot resistance gene in to susceptible vegetable *Brassicas* through sexual/somatic hybridization attempting embryo recue and protoplast fusion techniques. Previously, a single dominant locus *(Xca1)* for resistance to *Xcc* race 1 and 4 was reported in line PI 199947 of *B*. *carinata* [11]. The involvement of single dominant locus for resistance to Xcc has also been reported in other alien *Brassica* species viz. *B*. *rapa* [12], *Brassica napus* [35] and *B*. *nigra* [36]. However, some polygenes also played a role in controlling *Xcc* resistance loci in *B*. *rapa* [13–14].

### Mapping of black rot resistance

Information regarding genetics of race-specific resistance and location of resistant gene provides vital assistance in marker assisted gene pyramiding for development of durable resistance. The rapid identification of putatively linked markers viz. ILP (At1g70610, At1g71865) and SSR (Na14-G02) was facilitated by using BSA approach as used earlier by several researchers [31, 37–38]. Bulk segregant analysis (BSA) using microarrays and extreme array mapping (XAM) have recently been used to rapidly identify genomic regions associated with the phenotypes in multiple species by Becker *et al*. [39].

The level of polymorphism (15.19%) of microsatellite primers was found to be low in *B*. *carinata*, however this could have been low estimation because of the use of agarose (3%) gel, which can be further increased by the use of high resolution gel electrophoresis such as metaphor and PAGE. In spite of the fact that ILP markers are cross transferable from one species to another, their information in *B*. *carinata* is very less [18]. The ILP markers derived from B genome of *B*. *juncea* were found to have high percentage of cross transferability rates (93.75%) in *B*. *carinata* which confirmed the already available information that ILP markers have high transferability rates among related plant species. However, Yadava *et al*. [40] reported lower level of cross-transferability from ‘B’ genome derived STMS markers as compared to those derived from ‘A’ and ‘C’ genomes of *Brassica* species. 95% ILP markers of cowpea (*Vigna unguiculata* (L.) Walp.) were also reported to be transferable to other *Vigna* species by Gupta *et al*. [41]. This high transferability of ILP markers may be due to the conserved nature of exons in *Brassica* species. These findings are also in line with those reported in rice [26], foxtail millet [42] and Tomato [43]. The usefulness of ILP markers across species and genera is mainly because of their highly conserved exonic and variable intronic regions which decrease their developmental cost and increase utility. Across-species transferability variation in *Brassica* genomic SSR and ILP markers developed earlier that harmonizes with the existing evolutionary relationship among the *Brassica species* and genera. High transferability of these markers to *B*. *carinata* (93.75%) suggested their utility in these large genome amphidiploid species in which no sequence information is available. It reduces the cost and efforts required for marker development and enrich the resources for various genotyping applications in these species. Therefore, an elaborate and intensive polymorphism survey using a large set of such markers for construction of high-density genome map of this specie is required. The first sequenced plant species *A*. *thaliana* shares a common ancestor with the family Brassicaceae and provides a good opportunity in understanding the genome structure and evolutionary pattern.

Although systematic efforts have been made with the map-based cloning of resistance genes against blackleg disease [44], but there has been no such advancement in case of black rot [2]. In the present study, the resistance locus *Xca1bc* was found to be flanked by ILP markers At1g70610 and ILP At1g71865 with close linking to the former. The SSR (Na14-G02) marker was found to be located on B-7 linkage group in conserved block E of *B*. *carinata* [18], whereas ILP (At1g70610 and At1g71865) markers were found in conserved block E of *B*. *juncea* [30]. The nomenclature of linkage groups corresponds with the designation of each linkage group in the B genome of *B*. *juncea* [30, 45]. The organization of *B*. *juncea* linkage groups based on the genomic blocks was identified by Schranz *et al*. [46]. However, it was hypothesized that black rot resistance probably originates from the B genome [11]. These flanking ILP markers will be more useful with great efficiency in marker assisted alien gene introgression into *Brassica oleracea*. Previously, Tonguc *et al*. [47] identified RAPD markers associated with black rot resistance in *B*. *oleracea* breeding lines derived from *B*. *carinata* accession PI 199947 and found that all associated markers significantly deviated from the expected 3:1 ratio. Soengas *et al*. [13] identified one major QTL on A06 and two additional QTLs on A02 and A09 controlling the resistance to most of the races of the pathogen. Artemeya *et al*. [14] also reported QTLs against different four *Xcc* races (2013) in *B*. *rapa*. Molecular mapping of agronomically important traits has also been started recently after publication of first linkage map of *B*. *carinata* by Guo *et al*. [18] who mapped the loci for petal colour (Pc) and anther colour (Ac) on linkage groups B6 and C9, respectively. They also identified four QTLs for seed colour which were related to the C genome of *B*. *carinata*. Mapping of QTLs for flower development and construction of a dense genetic linkage map in *Brassica carinata* was carried out by Zou *et al*. [19] in a doubled haploid population based on DArT-Seq^™^ markers.

## Conclusions

The conclusion from the present study, therefore, is that resistance to black rot disease in *B*. *carinata* is governed by single dominant gene. ILP markers provide a novel and attractive option for marker assisted selection due to high cross transferability and cost effectiveness. Resistance gene is flanked by two linked ILP markers viz., At1g70610 and At1g71865 which will be a vital practical application in marker assisted selection with high efficiency. Therefore, to identify some more closely linked markers, this study has to be continued further by employing more DNA based markers to map the black rot resistance locus (*Xca1bc*) present in the resistant source‘NPC-9’ genotype of *B*. *carinata* and validation of these flanked ILP markers would be required in other potential resistant sources. Hence, search for new gene(s) in alien *Brassica* species and identification of tightly linked molecular markers would be of immense importance in the context of developing pre-breeding black rot resistant genetic stocks/lines in C genome *Brassicas*, especially in Cauliflower, Cabbage and Broccoli through marker assisted backcross breeding.

## Supporting Information

S1 Table Mean monthly weather data during experimental period. (PDF)

S2 Table List of SSR markers used in study. (PDF)

S3 Table List of ILP markers used in study. (PDF)

S4 Table List of polymorphic markers. (PDF)

## Abbreviations

SSR: Simple sequence repeats
ILP: Intron length polymorphic
Xcc: *Xanthomonas campestris* pv. *campestris*
DAI: Days after inoculation
