# Supplementary material for: Genetics and Molecular Mapping of Black Rot Resistance Locus Xca1bc on Chromosome B-7 in Ethiopian Mustard (Brassica carinata A. Braun)
Source: PLoS One. 2016 Mar 29;11(3):e0152290. doi: 10.1371/journal.pone.0152290 (PMC4811439; doi:10.1371/journal.pone.0152290)
Supplement: S1 Table — (PDF) [file pone.0152290.s001.pdf]

S1 Table: Mean monthly weather data during experimental period

Year	Month	Average Maximum temp. (ºC)	Average Minimum temp. (ºC)	RH1 (%)	RH2 (%)	Rainfall (mm)	Sunshine (hr)
**2010**	Oct	32.1	18.7	88.0	47.3	0.7	6.2
	Nov	26.6	13.4	92.7	48.3	0.4	3.3
	Dec	21.1	6.1	89.5	45.0	0.0	3.0
**2011**	Jan	18.1	5.3	88.1	46.4	0.0	3.8
	Feb	23.1	9.5	91.6	47.7	1.8	5.4
	March	29.2	13.0	85.1	38.5	0.1	6.9
	Oct	32.8	17.5	82.6	36.5	0.0	7.0
	Nov	28.8	12.6	86.5	34.8	0.0	4.2
	Dec	22.6	5.7	91.5	42.9	0.0	3.2
**2012**	Jan	18.6	5.6	91.1	54.1	0.5	3.3
	Feb	22.6	7.9	76.3	32.4	0.0	6.6
	March	29.8	12.6	75.6	25.1	0.6	6.7
	Oct	32.6	16.1	87.6	41.9	0.4	7.3
	Nov	27.3	9.9	89.0	35.0	0.0	3.4
	Dec	21.7	7.5	84.2	49.2	0.3	4.2
**2013**	Jan	18.0	4.7	92.0	65.5	1.3	3.7
	Feb	22.1	9.6	91.6	52.1	3.7	5.1
	March	29.9	13.7	87.0	35.2	0.4	8.1
	Oct	31.5	19.2	93.6	56.8	3.5	5.4
	Nov	26.9	9.9	90.9	48.2	0.0	4.9
	Dec	22.4	7.1	93.7	55.8	0.2	4.1
**2014**	Jan	18.6	6.8	96.6	66.5	0.6	2.3
	Feb	21.4	7.5	95.9	63.0	2.3	4.4
	March	27.2	12.7	90.0	48.0	2.0	6.4

Highlighted text indicates phenotyping period against Xcc race 1.
Source: Division of Agricultural Physics, IARI, New Delhi-110012
