# Supplementary material for: Genetics and Molecular Mapping of Black Rot Resistance Locus Xca1bc on Chromosome B-7 in Ethiopian Mustard (Brassica carinata A. Braun)
Source: PLoS One. 2016 Mar 29;11(3):e0152290. doi: 10.1371/journal.pone.0152290 (PMC4811439; doi:10.1371/journal.pone.0152290)
Supplement: S2 Table — (PDF) [file pone.0152290.s002.PDF]

S2 Table: List of SSR markers used this study

S. No.	SSR primers name	Foreword primers	Reverse Primers
1.	SSR:Na14D07	GCATAACGTCAGCGTCAAAC	CTGCGGGACACATAACTTTG
2.	SSR:Na10-D07	CTACTTTGATGGACACTTGCC	TCTGAAGTTGATTAGTCGGTCC
3.	SSR:Na10-D09	AAGAACGTCAAGATCCTCTGC	ACCACCACGGTAGTAGAGCG
4.	SSR: Na12E05	CGTATGTTTGTTCCACCTGC	ACTAGCAACCACAACGGACC
5.	SSR:Na12-D08	ACGACGATTCAACTCATCTTC	TTAACCAACTTCGCTTTTTG
6.	SSR:Na12-C01	CCAGGTTACTGTTAAAGAATAAGAGAG	ATCGTCTGCGAGTCTCCTTG
7.	SSR:Na14-G02	TTCCCTTTATTGAGCAAGCTG	TCCCGGTCGCTAAGATATTG
8.	SSR:Na12-A02	AGCCTTGTTGCTTTTCAACG	AGTGAATCGATGATCTCGCC
9.	SSR:Na12-G05	CCGATCATACCTTTTACTCTAGCC	GATGTTCCTCTCGGTGATGC
10.	SSR:Na12-H07	GCGGCATTAGTTGTCAGTCC	TCGTTGATTTACATACATGCG
11.	SSR:Ni4-C11	ATAAGGCCGATGTTTCCTCC	CACAGTTGCTTTGGATCTCG
12.	SSR:Ni2-D10	GATGCCCCAAATCTGTTACG	CAATTCGTGAAAAATAGCCG
13.	SSR:Ni2-F07	ACAAACAAAGCCTCCCAACC	TCACACAACTTGTTCAATCTTGC
14.	SSR:Ni2-A09	CGCGAGTAAATCAATGTGAATC	CGACCCACCAACTCACTAAC
15.	SSR:Ni4-A10	CAACGTCTCCCTCACAATCC	TCGCCTCACTCTCAATCTCC
16.	SSR:Ni2-D08	TTTAGGGAAAGCGAATCTGG	ACAACAACCCATGTCTTCCG
17.	SSR:Ni3-C08	CCCTAACACGGTGTCAACAG	GGCAGAATCATCGAGAGGTC
18.	SSR:Ni2-E05	CTCGTCTCAGGGATTATGTCG	CAGACAGAGGATAGACCGAACC
19.	SSR:Ni4-F09	CTGTTATGCAAGGTCATCGC	TGTTCCAGGTGAAGAAACCG
20.	SSR:Ni4-C02	TCCCTTGTCTACTTGCGACC	ACCCTTGTTCCCTCATCTCC
21.	SSR:Ni4-A03	ACACAGAAACATCAAACATACC	GGACCGGTTTTATTTGTTCG
22.	SSR:Ni4-F08	GAGAAAGAAGCAAACACAAAGC	TCTCTTTCTTCTCGTTGCCG
23.	SSR:Ni4-H04	CAAGAAAGGGTATTGCGTCG	TGTTTTAGAAATGGTATGCCCC
24.	BRAS61	GCAGCCTTCAACTCCCATAGA	TGGGTTCGAGCAGGGTTC
25.	BRAS116	TTTCCACCACCACCGCCATT	GGAAGCAGATGGAGACGGAG
26.	CB10080	GCCCTCAACCTGTAAAGT	TTGTTGGTGTGTGAATCATA
27.	CB10278	TGAAGAAGCTGGGACAAG	CAATGCAATACAGCACCA
28.	CB10079	TACAGGAAGATTAAATGCC	ACCGTTTGTCAGTGTCATAG
29.	BRMS36	GGTCCATTCCTTTTTGCATCTG	CATGGCAAGGGGTAACAAACAT
30.	BRMS46	TTGGCCTTGCTATTACGAGCTG	ATGCGCAAACCCTAATTTTCAC
31.	BnGMS044	GCGAAGATGAATAGTCGAAC	CTCTGTTTCGTCAAGCTACC
32.	BnGMS593	TAAAGTAGGCTGGTCGGTAG	GTCTATGGTAAGGGCACAAA
33.	BnGMS013	CAGTAGCCACAGAAGGTTTC	TTCAATCATCAGACTGCAAA
34.	BnGMS091	ACGCATTCTCTGAATTGACT	GAGCAGTGAGAAAGTCTTCG
35.	BnGMS 213	GTAGTACGGAGATGCGTGAT	AAAGAACGAGTTGACTTTCG
36.	BnGMS440	TCAATGTTTATTCCACCCTC	CTTCTTCCGACAAGTCTTTG
37.	FITO 361	GTTAGAGCAGCAGTTTCCC	TAGCCATTCACAACCAAAG
38.	SSR:Ni2-A02	GAGTGGAATCTTGCTACTGTCG	AAGGTCTGTGGAAATGACAGG
39.	SSR:Ni2-A08	AGAATTGGGATTTTCATTGAC	TCGTCTTCCAGCTTTCGTTT
40.	SSR:Ni2-A12	ACGATGGGTTCTTCTTGTCG	CAAGAAACTTTCGAGGAACCC
41.	SSR:Ni2-B01	AAGGAGATTGTTTTTGGGGC	AAGACTAATAAACACACGGCG
42.	SSR:Ni2-B03	ACTTCTTGCCCTCCTCACC	AAATACTCACTGCAATACCCAGG
43.	SSR:Ni2-C09	ACGGAAGAAATCCAACCTCG	TATGCTTGGAAATGGTTTGG
44.	SSR:Ni2-C12	ACATTCTTGGATCTTGATTCG	AAAGGTCAAGTCCTTCCTTCG
45.	SSR:Ni2-D07	ACCAAAGCTGATCTCCAACC	ACTCTTCGAATTCTTTTCC
46.	SSR:Ni2-E04	TTGCTGAAGACGAGACAACG	TTATGTTCGTTTCCGGTTCG
47.	SSR:Ni3-E06	CTACCCCTCTGCATCTCAGG	TTACTTGCTTGTCTTGGGGC
48.	SSR:Ni3-G04	ATACTCGGGATAGGTGTGCG	CATGTGGCAATCCTACATTTAC
49.	SSR:Ni3-H07	GCTGTGATTTTAGTGCACCG	AGCCGTTGATGGAATTTTTG
50.	SSR:Ni4-A09	AAAGGGCGAAGAAGCAGC	TTTCTTCCATTTGACCGACC
51.	SSR:Ni4-D01	TGAGCCACAAATTTGCTCAG	CAAAGGCGTAGAATAGAATTG
52.	SSR:Ni4-D10	ACATGCGAAAGGGATTTGAC	TGCAAGTGAACTCAAAACAAAAG
53.	SSR:Ni4-G02	TTGGTGTCAGAAACAACG	ACACACGACGGATCTCTGC
54.	SSR:Na10-B10	GTCGGGTTTGAGTGAGTTGG	CATCGCAGATCCTTCTCTCC
55.	SSR:Na10-C01	TTTTGTCCCACTGGGTTTTC	GGAAACTAGGGTTTTCCCTTC
56.	SSR:Na10-D03	ATGATTTGCCTTGAAATGCC	GATGAAACAATAACCTGAGACACAC
57.	SSR:Na10-E02	TCGCGCATGTAATCAAAATC	TGTGACGCATCCGATCATAC
58.	SSR:Na10-E08	TCGGGGTTTGTTGTGAGG	GAGGAGGATGCTAAGAGTGAGC
59.	SSR:Na10-F08	AAACTTGCTTTCGAGGATGG	AAACCAGTTGACTCCATCGG
60.	SSR:Na10-G01	TGTGTGGGGGAGAGAGAGA	AACCGACCCGAACTAAACG
61.	SSR:Na10-H03	GAGCTGGCTCATTCAACTCC	CACAATTTCTCAGACAAAACGG

S. No.	SSR primers name	Foreword primers	Reverse Primers
62.	SSR:Na12-A01	GCATGCTCTTGATGAACGAA	GCTTCAACCTCTCAATCGCT
63.	SSR:Na12-A07	TCAAAGCCATAAAGCAGGTG	CATCTTCAACACGCATACCG
64.	SSR:Na12-A10	TTGAACTCTAACAAAGATTCCGC	TTTTTCGTTTGGATGCAG
65.	SSR:Na12-B01	CCAAGTCTCATGTTGAATGAAAG	CTCCATAGCTTCTCATGCCC
66.	SSR:Na12-B05	CAAATATCCGTCATCGGAGC	CCTGCGGGATATTGAAGACC
67.	SSR:Na12-C07	ACTCAACCCCACAAACCTG	AGTTCCCCGGATCCGATTAG
68.	SSR:Na12-C08	GCAAACGATTTGTTTACCCG	CGTGTAGGGTGATCTAGATGGG
69.	SSR:Na12-D07	GAATCCAACGGATCAGAAGC	GCGTTCCAGAGACTCCTCC
70.	SSR:Na14-A06	GCCTGTCTTCTCCTCCACAC	ACATTGGATTATGCCCGTTG
71.	SSR:Na14-B05	ACTGCTTTGGCCTTGTCTTG	AGGCCCTTGATCGGTAATG
72.	SSR:Na14-C12	CACATTTTGGTTCAATTCGG	TACGACGCTGGTTTCGATTC
73.	SSR:Na12-E06A	TTGGGTTGACTACTCGGTCC	CCGTTGATTTGGCTAAGACC
74.	SSR:Na10-G05	CGCATGTTCCCACTACGATA	AAAACTAATTGACAGATGTGTTCTC
75.	SSR:Na12-E01	ATTCCATGACTCCATTGTC	AAATCCCTTGTCTCTGTCG
76.	SSR:Ni2-E07	GAGCGAGTCGATTACTTTTGC	GAATGGATTTCCGATGATGG
77.	BRAS063	GACGCTCATTTCACTTC	TCCTAACTAACATCATTTTGC
78.	BRAS004	GTTCATTTGACGGCAACTCTC	CGCCAACGAAGCAGGTC
79.	BRAS119	ATGAAAATATAAACGCTGCT	TACCTTGAGGACCTGCGACT
80.	BRAS107	TGAAACCACCATCATCGGAG	CTGCGTTCGCCTAAAGTAT
81.	CB10587	TTGTGTTTTGCCTTCTGA	TTTGCGCACAAACAATAA
82.	CB10403	CCACACATGAACCCTGTT	AGGACCAAGATTGGAAGC
83.	CB10003	ACGGTGCCGAATCTCAACG	AAATGGGTCACAGCCGAGAA
84.	CB10065	CGGCAATAATGGACCACTGG	CGGCTTTCACGCAGACTTCG
85.	CB10439	ACCTCGAAGGGTATCTGC	CGTGCAATTTCAACAACA
86.	CB10092- B	TTGATCCGAAATTCTCTGG	AGGCAAGCAATAGATAAAGG
87.	MR153	AAC AGA AGA CAT GCG GAA AC	ATT GAT TTG AGC CGG TAA AGA
88.	MR113	TCT ACC ACA TAG CAT CCT AGC	TGG GTG ATT TGA GAA CTC ATA
89.	BRMS-2	GATCTTCTCTCCAAAA	TCCAAGCTAAATTACG
90.	BnGMS277- A	GTAGAGGATGATAATTGCGG	ACACGTGCTATCTCGTCTCT
91.	BnGMS380	TATAGCCACGAAATTCAACC	AATAGCAGTCAACATGCAAA
92.	BnGMS 345	TGGCTAATCAAATGACAAAG	GAACGGATAACCGTCTAATG
93.	BnGMS289	CATTACAAACTCAGCGTCAA	CAGGACACTCGGTTATCAAA
94.	BnGMS426	AGCCAAGCTCAAAGTTAATG	CGTAACAGTGTTGAAGCAAA
95.	BnGMS256	ATCATCCAATAACCAAACCA	TATCTGCTTGTTTCTCCCAT
96.	BnGMS292	TCCGCTTTCTATTCTCTTTG	AATCCAACTATCTTCAGCGA
97.	BnGMS334	GATGAATGATGATGCCTTCT	CAAGCCGCAGTTATTCTTAT
98.	BnGMS323	TTGCTAATATGGCCTTCAAT	AGCTTTGCTTTCAAATCTGT
99.	BnGMS567	GGCCTTAATTGTGCTAAATG	CTCTCTCTTCCTCTGAGCTG
100.	BnGMS277- B	GTAGAGGATGATAATTGCGG	ACACGTGCTATCTCGTCTCT
101.	BnGMS508	TTTATCGACCCAGAAAGAGA	AGAAGAGAGAGGAAGAGCGT
102.	BnGMS392	ATGACAAAGGCCTTCAATAA	AAAGAGCGATTGGAACACT
103.	BnGMS298	GGTGCATACTACTTAAGCCC	AGCATGGTACGATTCAGTTT
104.	BnGMS148	GCCCAAGAGAGACACAATAG	GATTGGGTGAGAGAGAGTGA
105.	BnGMS679	ACAGAGAGAATGAGAATGCG	GAAGAAGGACGCAATCATAG
106.	BnGMS085	CCTCTTCCTCAAATCTCCTT	ACGAACACCTTTGAAATGAC
107.	SSR:Ol13-E08	TTCGCAACTCCTCCTAGAATC	AAGGTCTCACCACCGGAGTC
108.	SSR:Ol10-B04	ATCTTCCTCCACGTTCATGC	CGAATCTTGAAGTTCTGACCC
109.	SSR:Ol12-D01	CATTCAGCACTCGCTCATGG	TCTCAGGCACTCTTTAAAGC
110.	SSR:Ol11-H02	TCTTCAGGGTTTCCAACGAC	AGGCTCCTTCATTTGATCCC
111.	SSR:Ol11-D12	CCTCCACCGCACTCAATTAC	TGGAGAAGTTTGGGACATTTTC
112.	SSR:Ol10-B06	GCTTTGGCTCGTGTAAATGG	CATCTTTGGTTGTGTATGGTTAGG
113.	SSR:Ol10-A10	CCTGAAGATAGGTTTGCTTCC	ACAAATGCAACTACTAAATTGTCG
114.	SSR:Ol10-D08	TCCGAACACTCTAAGTTAGCTCC	GAGCTGTATGTCTCCCGTGC
115.	SSR:Ol11-H05	GACGGATTCCTTGTAAGTGG	GGTGTTTTTAGGGCGAGCTC
116.	SSR:Ol11-E03	GCTCTCCCAGTGAGAATCAC	GAAAACCAATCCAGTGCCTG
117.	SSR:Ol12-A04	TGGGTAAGTAACTGTGGTGGC	AGAGTTCGCATACTCTGGAGC
118.	SSR Ni1-A04	TCCTCCTACTTTGATACTTGC	ACGTCAAATACTTCACTGCC
119.	SSR Ni2-A01	TGCTGCTACAGACAGTGTTGG	AAAGGCTACACACTCATGAAACC
120.	SSR Ni2-A06	TGGCTCCTTTATGGTCTTGC	AACCATGTAGCTTTGGACGC
121.	SSR Ni2-A07	GGAACCCAACAAGTGAGTCC	AGAGCTTGAGACACATAACACC
122.	SSR Ni2-A10	TTTTGTTGCGATCTTGAAGC	ACACTTCCCAATGTCAAACG
123.	SSR Ni2-A11	AACAAACAAGAGTCGAATACGG	AATGCCCTCTAACTGAGCCC
124.	SSR Ni2-B02	CGCTGCAATTATACGAAAGC	CCTCATGCTCTCCAAAGACC
125.	SSR Ni2-B07	AGAGATTCAAACCGAGTGCC	GGGGCTAGCTTCATCATCC
126.	SSR Ni2-B08	TCACAGGATTGGGATCTTCC	ACGTATTGATATATGGATCGAG
127.	SSR Ni2-C01	GAGTATGAGAGATGGGAATCCG	GACTGAGCAGCTTGGAGACC
128.	SSR Ni2-C03	CGTAGAAGATGAACTCGGGG	CTCTTTCAGCTACTGCTGCG

S. No.	SSR primers name	Foreword primers	Reverse Primers
129.	SSR Ni2-C06	CACTGGGATACAAGCCCTTC	ACAATTTGAAGTACAAAACTCTC
130.	SSR Ni2-C08	TTACCTACTACGGCGAACGG	AGGAGCTCAGATCGTCAAGG
131.	SSR Ni2-D02	AACATAGCCAAAAGCATGAGC	ATTGATTGAGATCATGCGCC
132.	SSR Ni2-D03	CGTATGTGAAAAATAAATGG	TTGAGCTTGAGATCATCCCC
133.	SSR Ni2-D06	GGGGAAGAGAGAGAGAGAGAGAG	ATTTGTAGCCCTAGTGGCCC
134.	SSR Ni2-D12	GAGATGAGGATTTGCTTTTGC	ACAGTATGAGAGAGAGAGAGAGAG
135.	SSR Ni2-E03	TGGATTACAAGATTTGCCTGC	CGTGTTTCCTCGCTAATTCC
136.	SSR Ni2-E07	GAGCGAGTCGATTACTTTTGC	GAATGGATTTCCGATGATGG
137.	SSR Ni2-E10	ACTGCTTCAGCACGACCC	CACATGTAAACTCTCCCACAGG
138.	SSR Ni2-E12	TTATCTGCTTGTCTTGGGGC	AAGGAAATCGTCTCACTTGG
139.	SSR Ni2-F01	CGTATGTAGAGAGAGAGAGAGAGAGAGA G	AGAACCGTTGAGGTGCTGTC
140.	SSR Ni2-F03	CTTGCTCCTCCAGATTCGTC	TTTTCAGATATAAGGAAAATTACTC
141.	SSR Ni2-F04	TTTCTTCTTAACCATCGGCG	TCTTCCTCTGCTTCTGGTGC
142.	SSR Ni2-F06	AAGCTAAAAAGCCAAGCAAGG	CTTTTTCATCAAACCGCTCC
143.	SSR Ni2-F11	AAAGGGTTTCAATTTCACGC	GGGAAACATACTCACCACGC
144.	SSR Ni2-F12	TGCACAAGAACGAAATGACC	ACGAATATCTCTCTCTCTCTCTC
145.	SSR Ni2-G06	TGGATACGTCACTGTCACTGC	GAAACTCCGTCGCTATCTCG
146.	SSR Ni2-G08	TCGACCAACAGAGAATGAAGAG	TTTCCCCATGAACACATTTC
147.	SSR Ni2-H03	TTTGAAGAAACAAAAATGGCG	TCATCTTCCCCTCTCATTCC
148.	SSR Ni2-H05	ATTTCGAAGCTTTCACCACC	CTCATGGTGCTGAATCAACC
149.	SSR Ni2-H06	CATCAGATCCGACGAAATCC	TCCTTTGGACTGTGAAAAACG
150.	SSR Ni3-A05	ATCGGACAGACTCCTCTTGC	TACCCCCTCTGCATCTTAGG
151.	SSR Ni3-B07	GGAGAAGAGGAAGAAGAAGCC	CGACTTCTAGAGGAACCCCC
152.	SSR Ni3-C05	TTTCGTGCTTTGGTGTGAAG	TCCCCAAATCGAACCATAAG
153.	SSR Ni3-D03	ACCGGAGACGAAACTACCG	CCTCTTCGACGTTTTTGGTG
154.	SSR Ni3-D04	CACGTTTACTTCTCCAGCCC	GCCCATCAAGAAATGGAGAG
155.	SSR Ni3-D09	GCTGATGACAAAGGGGGTAG	AAAAGAGGACAAACAGCCCC
156.	SSR Ni3-F01	AGCCGCTAAAGAGAAGGTCC	CGCTTTCAAGCTCTCTCCC
157.	SSR Ni3-F02	TCCAACTCAATGGAAGAGGG	ACCATTGAAACGTTGAACCC
158.	SSR Ni3-G05	AGGAAGCATTTGCGCTAGTC	TCTACAACCACAACGTCCAAG
159.	SSR Ni3-G07	CACTCTCTCCGCCATTTTTC	CTTGAAGCGTTAAAGCCGAC
160.	SSR Ni3-G08	CTTAACCAGCCCACACATTG	TTTTGGGTTATGGGGTGAAG
161.	SSR Ni3-H02	ACTGCCCTTGCCTTATCTTT	TTGAGGAACAAATCCTTGGC
162.	SSR Ni4-A02	AGGACCACTGGGATACAAGC	ATTTGGAGCTGCGTACTTCG
163.	SSR Ni4-A04	ATGTGGTCTTTCCCAGTTGC	CATCCTCTGCTTTAGTGGGC
164.	SSR Ni4-A05	AAGGGGTTTGTTGTTGTTGG	GGCGCATTAGATTGTCTGG
165.	SSR Ni4-A06	ATCTTTGGCTTCACGATTGG	CCTTCTTCTTAGCATCTAACTCCC
166.	SSR Ni4-A07	TTATCTGCTTGTCTTGGGGC	AGACACTCTCACCCCTCTGC
167.	SSR Ni4-B03	ACTTTCTTTACATTCTAATCGC	GCTGTTTCTGTGGAATTGTCG
168.	SSR Ni4-B04	TAGGCGGACTTGTTGATTCC	CATGATTCAGAACAAGGGTGC
169.	SSR Ni4-C08	GAGAGAGACGGTTTCTTGCG	TCCATAATATTCTACAACTCATCACC
170.	SSR Ni4-C09	AGCATCAATCTTTTGCTCTGC	TGCACACAAACTCCTTCTCC
171.	SSR Ni4-C10	AGATGCTAAAGCGGATCACC	CACTTGGTAACTCTATGGATGCC
172.	SSR Ni4-D04	TTTAAAGTGTGTTTCACAAACTGG	GAGTTCACAGGGGCTACAGG
173.	SSR Ni4-D08	AGAGATGCTAAAGTGGATCACC	CGGGATTTTGAAGACCTGC
174.	SSR Ni4-D09	AAAGGACAAAGAGGAAGGGC	TTGAAATCAAATGAGAGTGACG
175.	SSR Ni4-D12	ACCACCATCCACAGAGTTCC	GCAGGACAGACTGAAAGCG
176.	SSR Ni4-E01	CGAATATCGTTCGTTAATCCTACTG	TCCAGAGCGATTGGGTGTAG
177.	SSR Ni4-E03	GAAGGTGAGGAAACTGGTGG	AACCTCCTTCTACCGCAACC
178.	SSR Ni4-E08	GATTTTGAGGAAGCGGAGG	CAAAGCACTGAGAGAGAGAGAGAG
179.	SSR Ni4-E11	AAAGGGTTCAGAAGTCCTACCC	TGGCGTAATTTTTCTACCGC
180.	SSR Ni4-F02	CACTCGGAGAGATAGAGAGAGAGAG	TGGTACGAAGAAGTGAAGAGAGAAG
181.	SSR Ni4-F06	AGATTCGATTCAAAGTGTTGG	TCCCTCTAGATTTCTCCGCC
182.	SSR Ni4-F10	TATGTGTGTGTGTGTGTGCG	TCCGTTTGATTGGGTCTCTC
183.	SSR Ni4-F11	CGTAAGTTTCAATTGTCAACGG	TCGTACGAAACAATCAACGG
184.	SSR Ni4-G01	CTCAATCGCATGCATAATCG	CTCGAGGCGCGTTTTACC
185.	SSR Ni4-G06	TGACGGCTGAAGAAAATCAG	GTTTAACCTAAACCGAAAATC
186.	SSR Ni4-G08	ATTTGACGGACTCCTCTTGC	CACTTGGTAACTCTATGGATGCC
187.	SSR Ni4-G09A	CTCGAGGCGCGTTTTACC	CTCAATCGCATGCATAATCG
188.	SSR Ni4-G09B	AAAAACTGGACCCAATTCC	GGTTAGGTCATAAACCCAAAGC
189.	SSR Ni4-G10	AGACTGAAATATTTTGGGACC	CGTTCTTCAACTTGTTCATCATC
190.	SSR Ni4-G11	GCACCATCGAAAAAGGTCC	CTGGGTATGGTTGGAACAGG
191.	SSR Ni4-H01	TGAAAACACACACACACACACAC	AACCCTGATGTTGAAGGTCG
192.	SSR Ni4-H03	GATGAACAGCAACAGCTTGG	CAAAATGTCGTTTGTTAGTCTTGG
193.	SSR Ni4-H05	GAAAACACACCACCAAACCC	CCATAGAGTTCTTGTTTCTCTCTC
194.	SSR Ni4-H06	TTTCCATGTAAGGAATCGCC	CCTGTATACATAAACCTCTCTCTCTC C
195.	SSR Ni4-H08	ACCTCAGTTGCTTATCATCTA	AATTGGAAGGATTTGCGGAT
196.	SSR Ni4-H09	AGAGATTCAAACCGAGTGCC	GGGGCTAGCTTCATCATCC

S. No.	SSR primers name	Foreword primers	Reverse Primers
197.	BrBAC205	CCAAAAGGAACCACCTGTGT	AAATTAACCAACGAACGTGAA
198.	BoSF2969	TTGAAAGTCGACGAGAGGCT	GCCGAACTGCAAATTTAAGC
199.	BoE139	GAACGTAGAGACAAAAAGGAGAGC	CGTGGAGATTATCGCCGTTATT
200.	BrSF561	CAACTCCTAGCCCACTCTCG	GACAAAGAGATCGAGACGGC
201.	BoSF042	CGGCTTGACAGAATTGGACT	CGGCTTGACAGAATTGGACT
202.	BrSF189	ACGACTAGACGGTTGGGATG	AAGGTCCGAGCAAGAAAGGT
203.	BoSF2712	CCATCGATTCCGTCAAAACT	GCTTTGAGTGTGGTTTGGGT
204.	BoSF2713	CCATCGATCCCGTCAAAACT	GCTTTGAGTCTGGGGTGGGT
