# Supplementary material for: Genetics and Molecular Mapping of Black Rot Resistance Locus Xca1bc on Chromosome B-7 in Ethiopian Mustard (Brassica carinata A. Braun)
Source: PLoS One. 2016 Mar 29;11(3):e0152290. doi: 10.1371/journal.pone.0152290 (PMC4811439; doi:10.1371/journal.pone.0152290)
Supplement: S3 Table — (PDF) [file pone.0152290.s003.pdf]

S3 Table: List of ILP markers used in study

S.No.	ILP Primers Name	Foreword primers	Reverse Primers
1.	At1g02410	ATGGAGGTACTGTTCAACGCAA	AGGAGTCGCTGCTCCTCA
2.	At1g03180	TCGTCCTCTATATGCACCAGCA	CCAGGAGCTTCACGGATCA
3.	At1g03330	CGTGTTGTTGATCAGGACAAG	TACCTCACCACTGATCCTCTG
4.	At1g03910	CTGAAGCCTATCGACGTCC	TCATCCCGAAGCTCTTCC
5.	At1g05055	AGTGCCATTTACCATGCTCA	TGACTCAGAGGATTCTGGTCA
6.	At1g05720	GTACGTTTTCAACTCACCACC	TGAAGTAGGCTTGACCTTCTC
7.	At1g05850	GCT GTT GGT TTC TGG GAC TAC CA	CCA GCT GCA CCG TAG TTG AAG T
8.	At1g08780	ACATTAACACCTTCAGCCG	CGCACCATCTCCTCATC
9.	At1g09760	AATCACTTCGAGTACTGGATTTCAT	CGGCAATTTCTTCTATCGTCTG
10.	At1g10840	GAGACCTTCATGAATTACCAGG	CTTCAGTCTCCAGTTCGGTC
11.	At1g12840	AGCTTTGTGGAAGGAGTTTCGCA	TCGGTTAATGGCATTGAGTTGA
12.	At1g15370	CGTGTTGTTCGCTAATTCCGT	ATTCTCAGCTCCCAACTTAACC
13.	At1g16740	GAGGTCTCTGGATCGAGCG	GGTTCAGCTGGATGTTCTCCTT
14.	At1g18340	CTTCTCTCTTGTCAGGATCACTCTC	AACATACTGTTCCGGGCCATCT
15.	At1g19240	GCGAAATCGGCAGCTTCTTCTT	ACCCGTCTCTTAAGCGTATAACCCAC
16.	At1g20410	AGGCTCAAGGTGCGTCTG	CCTTTTCAAATGAATTCTCGGA
17.	At1g23180	GAAGCAGCCAGAGATCTCACTAA	TCGTCAAACCAGCTTCTTCTAGCT
18.	At1g26180	GGCTCGTTTACTTTCTCAGCAA	CGAGAAGGATGATGTCAGAACATA
19.	At1g28530	GACACAAGGCAAAGAAGTCGG	GGTAGCTGTTTCCTGGGAAGTG
20.	At1g28560	CTG ATA TGG CTA TGA AGG AAG CC	TGT TTG AGT TTA GCA AGC TGC TC
21.	At1g47570	CGGAAAGACATGAATGGTGCAG	CTTGAATCTGGAGCAGGCATTA
22.	At1g57680	TGCTCAGATCTTCCCCGTT	ATCCACGCATGCTCTCTACCA
23.	At1g58220	TGA CAC CAT GAC GAT ACT GAA G	TTG ACA TGC GCA ACA GCT T
24.	At1g60430	ATCCCTGTTCCTGGAGAAAC	CGTCAGTAACTTTGATCTTGTATCA
25.	At1g61780	GACGACGAAGATGGTGTGC	CATCTTGATGCACTTGCTGC
26.	At1g67170	ACAGGAGTACCAGCAGTGCA	CTCCTTGATAAGGGTATTGAGCTG
27.	At1g68310	GGTGGAACATTGTAGCATGG	ACGTTTGGATTCTCTAGTGCAG
28.	At1g68660	GGAGATTAGCTCTCGCACCA	GCGTTTTCTCTATAATTGGTTTGTC
29.	At1g70610	TGGGTTATCTTCGCTGCGTT	GTCACCAACAGTTTGAGAGTCGA
30.	At1g71865	TTGCGTCTCCAGATCTCAA	GATCTTGCAGCTTGAATGAGTGA
31.	At1g72380	TGCAGATCATGAAGTTTCCTT	ACGTTTCCGATAGTCATGCA
32.	At1g73530	GTCTTCTATCTCTGGTCCGAAGAC	TCAGTTGTACGGAAAGAAAGCC
33.	At1g75980	CGGTCTGAATCGAATACAGC	GTCCAATCTGCAGGTCTTG
34.	At1g78560	GCTGGCTTTGTGTTGCAG	CCTGGACAGCAACCAACC
35.	At1g79150	CATATATTGAACAAATCTCAGAGGAGAG	TTCAAACGACTCTTCAGCAGATA
36.	At1g80460	ACCAGCACCAGATTCATCAT	CAATGCACACTTTCACACTTTC
37.	At2g01940	CATTCTTGTGACTGTGGTCG	TGATGTTCAATAAAACTCTCCACC
38.	At2g07340	TCTAGAGATTCAAGCAAGCATGAT	GGTTCTTGCTGCAACAGCTC
39.	At2g07690	CTGATAGGAGCCAGTATGTTGA	AAATGTTGCTGTAAACATCTTGAC
40.	At2g15290	AAGGCAAAGCTCGCTCAG	TCAAACCTTTCACAACATCAGC
41.	At2g15430	AGATTGACTTGATTGAGAGCAG	GTATGCTTCTGGATCAACCAC
42.	At2g17710	AGAAGCAGATTATGGATGAGTCTG	AAGAGCAGGCTGGTTCAAGT
43.	At2g20490	GACGAAGATGTATCTTCAGTGCT	CCGCTTCTTCAACAAAACTCTCT
44.	At2g21620	ATAGCCGTTGATCATGGTCC	TCAACAACACGAGCCACACT
45.	At2g22000	TCTTCTCAACTCTCCATGTTCTG	GCGGTCTTGGTCTCCTTCT
46.	At2g23090	GGTGGAGGAAACGCACAGA	CTCAGCGTGCTCCCTGCA
47.	At2g26590	GTTGTCCCTGATGCACGA	TCTGCATCACCTTCAGCTCT
48.	At2g31490	CGGAATCTTCGGCATCGC	TGTGGTCTGCCTGCATCTTC
49.	At2g32520	TCCAAATCCAAAGAGATGACACT	CCCCACCATTCTTGAATCAC
50.	At2g33400	GCT TCG GTC TCT CTT CCA ACA	CAT CAG CGT CAT CTT CAT AGT CG
51.	At2g34860	CTCTTCGCAACCCTTCGC	GCTGGAGATAAGCACACACATG
52.	At2g36530	GCC ACT ATC ACC GTC GTT AAG GC	GGG CCG ATG ATG TTG TTC ACA
53.	At2g36930	GCGTAAACCAGAGACGGAGA	GAGTTGTGAGTGTGGCGC
54.	At2g37560	TGTTGTCATCAATGGCTACCT	GGTTTGGGCTGTGCTTCC
55.	At2g38130	TGGAATCTGGCTGTGAAGAG	TGAACCCGAGTCTCCCATATAG
56.	At2g40765	GGCGCCATATGGGTCGT	TGTCTTCTTAATCCAATCAAATGG
57.	At2g41530	ACTTTCTCCATCTACTTCCCTCCT	TACATACGCCAGTTCTTCCACT
58.	At2g43360	TCCAAGGACGCTGTCAATG	TGTTGCTTCTCAATCATGCC
59.	At2g44050	CTTCAGCTCCGAGCTCTTCA	GGAATTTAAGAGTCGCTCTTGG
60.	At2g45690	GGTGTGGAGAAACAGAGAGTATGT	GTCCCGGAAGGTCCAACA

S. No.	ILP primers name	Foreword primers	Reverse Primers
61.	At3g02420	GGCTTCCTTCTGATTATCTCTCTG	GGAGTATTCAAGAAGGGAGCGT
62.	At3g02860	GTACAACGAATCAGACCAGCCT	GAAGAATCCCTCTGGAAGAGGT
63.	At3g06483	CTCTCTGAGAAACCTGCCGT	CCGGAGCAGAACCATACTCTC
64.	At3g08690	GGA TCC TCC TTC TAA CTG CAG C	GAT GGT AAG AGC AGG GCT CCA
65.	At3g09050	GACAAGGTTGGAGAAGACGCC	ACTTTTCTCCAATCCGGTGAAG
66.	At3g10572	TGCTCTATGTATGAAGAGGCAGAG	CCACAACTTCCATGTACTCATCAA
67.	At3g12260	CACCATCATGGACATTTACAATCT	CAGCTCTTCCATTCCCTTGAA
68.	At3g13120	TCCAGAAACTCTCGATGAACCC	GAGGCACCCAGTATGATCTAAGC
69.	At3g14850	AAA TGG ACG GCC GGA TCT	GAT TGC CAT TGA TT(T/C) AG(G/A) CTA AG
70.	At3g15190	GCTTCTCTCAGAGCGTTTCTCAA	GCCCATCGAGTGCTTCCAA
71.	At3g17300	GCCTCCTGAGCTCGCTAACA	GGGGTCAGTCTCATTCATGTG
72.	At3g18600	AAATGTGGCGGAGGAG(C/T)TA	CCAGTATCCTATCAGCTTCATCGAT
73.	At3g20920	GGAGCCGAGAAGAAGAGGGTTA	GGGTTTTGGTAACACGATCACA
74.	At3g23980	CAAAGGGATCTTGATGCTTCA	TCCATATCATCTTTTAGTTGGTTGAC
75.	At3g43610	CTGACGTCTGGTGTTCACTGAAG	CTTAGATAACCCATATGCACAGACTC
76.	At3g45620	GTCGAATCTCCGCCTCTGAG	GTAGAATACGTTAGGATCTCCAGGC
77.	At3g47370	CACAAGATCAGAATCACTCTCTCCTC	GAAGAGATCGATCACACGCTTG
78.	At3g47610	GAGACCGGTTTGAATTTCGATCG	AGCTGCCACATCAGCATCAGATACT
79.	At3g48100	CAGAGAACATCTTGCCTCGTAT	AGCTGCGAGTAGATATCATTAGCT
80.	At3g48540	GCTCTTGCGTTTCGCTTCTTCTC	ACACCATTTTGGCTCACCAAACA
81.	At3g52990	ACC TCT TCT GGA AGA GCA GCC A	CAC CAC AGA TGC ATC ACC AAC T
82.	At3g53190	CTTCTCATACCACATCCCGACC	TGTTGCTTGGGAACACTATCCAC
83.	At3g54130	CCGATCTCGACGGGAAGGAGCG	GGGTCTATCTGCGCAGGCTCTGCA
84.	At3g54670	TCGGATGACTGATCTTTGTCG	CTTGACACGTACTGACTGAAGAGG
85.	At3g55005a	GAGATGATGGATCTCAAGACC	GAACTTCAAGAATCCTTCAAGA
86.	At3g55005b, c	CTTGAAGGATTCTTGAAGTTC	TCTCTCCTTGAATTACCACC
87.	At3g55430	GCTGGTGTCAGAGATGTTAAGG	CAGGTCATAGGCTATGATGTTAAG
88.	At3g55440	GTT CTT CGT CGG AGG CAA CT	TAT GGA GGG CTA ACC ACA ACC T
89.	At3g57650	GCCTCTGGGCATTCTCTTCTTC	CCCACAAGCCAATCAATATCAC
90.	At3g63420	AAGCACATGATCCTTGCGGAGC	CTTCAAACCACCGGTCCCATCC
91.	At4g00585	GAGTATGACTGGTGGGCCTAACTGT	GGTTCCAAAGTTCTTGCACCAGATC
92.	At4g01310	TCTCCTTCGCTTCTGCAGTCTT	TTCTTGATCGCTTTTAGCCGTT
93.	At4g01897	AGTACGGAACAAGAATGGGAAGAGT	GACCATAGAAGTGAGGGAAGCTATT
94.	At4g02425	GAGTGAGCCTGGGTACAGAG	ACCTGAACTGAGGTTTACTATCTGA
95.	At4g04190	GTCCTCCATCTCCTGTTATCCCTC	CCTGCTTCAGTGACTCCAGTCTTT
96.	At4g07666	AATGACATAAGTCTTGAKGAACAACT	ATAGTGCATYRGGTGGCTCT
97.	At4g10050	AGACTCTCCTCCAGCTATTGTGC	GATAGAGACACGGGCTGAATCAA
98.	At4g10180	GTTGATGCTCGTGCTATTGGTTAC	GATTAGCAGGTGTTGTCGATCCAA
99.	At4g10930	ATGTGAAGAACTATACGGACCTGG	GGTTTTAGTCACTGCCCATCTGTA
100.	At4g11790	CAATAGCCAGACTGGATCTTTTAGC	CTTCATGAACCACAGTAATACCCTT
101.	At4g13720	CTCGTCTGGCTGCTCTTCA	CTTCATGGCCAAGCTTCTC
102.	At4g14110	AAGCTAAAGATATGGTTGCTGC	AGATGCTGTATCCACTGTCCA
103.	At4g15520	CTGCGTTTCTTCTCGGTAATGAG	GTAACATTCAAAGAGGCAGTGCC
104.	At4g15530	GGA AGA AGC GAA GGC AAC AAG	GTA TCC ATC ATA CCG GGC ATT GA
105.	At4g15910	ACTCTCCGGTGCCGTTAAATCT	CTCTCTGAGCTCAGCTGGATCA
106.	At4g16060	CATTGGAAGGCAAACCTTACACC	CAGACCCTGTGCTGCTTAAATACTC
107.	At4g16180	CATCACAGTGCTTTCCTTGACGTA	AACCTCTAGTGCAGTTCCAGC
108.	At4g16280	ATGGGGAGAGAGAACGCATAG	TCATCACGCATGAGATAGACATC
109.	At4g17050	CGA ATC CAA CAC TCT CTC CTT C	GGA GGT AAA CCT GAA CTT GAC ATT
110.	At4g18150	GGGTGGTGTTCCGGCGTCT	TTGTTTCCGCCACCTCGAG
111.	At4g18230	CATTGCTGCTGCTACAGATAACATG	CGAGGATACTTCTTATGCAGTTGTG
112.	At4g18400	AAACGTCTACTCCTCCGGCTACA	TCTAACCTCGTCCTTTCTCTTTTGG
113.	At4g18740	CCAATCATCTTCATGTTCGAGG	CGTCAAGAATAGCTTTGGTCAG
114.	At4g19140	ACTACTGGGCTTCTGTCTTCAAGGT	CCAATCATCTTCATGTTCGAGG
115.	At4g20150	CAA TGA GCG CCG TTA CTT TG	TCC ATA ACT AAT CCC GAT CTT CAT
116.	At4g21720	ATG ATA CCG CAG CAA TGG ACG	GAG TCA CTA TCG GCA TCA CAT CC
117.	At4g23860	GGC ATC TGG TGT TTT CGA GGA	TTG CCC GGG AGA AGC TTA CA

S. No.	ILP primers name	Foreword primers	Reverse Primers
118.	At4g24680	TAG CAC GAC AGG AGA TCG AAG	GCA TTC AGA GAC GAT TTA CTG CC
119.	At4g25140	CAA GTC TAG GCA GAT TGC TAA AGCT	CAG CTT CAT CCT TGC ACT GTC
120.	At4g26240	CAG CAG TTC ATG TCA CCA TGG GA	AGC AAG CCA TGA TAG CGA TCC A
121.	At4g27490	TGA TGT ATG ACC TTA TCA CAG CTG T	CAA TCA CGC ATG ATC TCT CCA A
122.	At4g29660	AGT TCG AGA GAT TTG CTG TTT GGG	AGC ATC TGA AGC AAT AGC CTC ACC
123.	At4g30790	AGC CAG TCT GAG CAG AGA GTT GG	AGC AGT TCT CCA AAT GCG CAC
124.	At4g31130	GCC WGC GTC TGG GAC TCC AAT	CAT CCC TGG AGG AAG AGT TYC CAT C
125.	At4g33100	GAT TTG CGA AAC GCT TAC CAC AA	ACC TCC AAA ATG CGA GTG AGA AG
126.	At4g33140	CCA TTC AGT CTC AGA ATA CCA TGTC	TGT CTT AAA GAA TTC ATG AAC ACG T
127.	At4g33925	TAA CCG AAG AAC AGC TCT CAA TGT C	GAA AGC TTC AGA TCT TTG AGA GCA
128.	At4g34140	AGG ATG GTC GTT ACT ACA AGC ATG	GGA ATC CAT TCT CCC TCT TCA AG
129.	At4g34412	GGC TGC TGC TCA GAA AGC ACT	TCC GGC AAA CAA TGG CAT C
130.	At4g34700	CTT CTG ATC TGC GCG AGA AGT	GCA ATC AGT TTG TCG ATT CTG TC
131.	At4g36960	TTA GGG AAC AGG ATC TTG GAA GT	TGT TGT GCC TCC CAG ATC AT
132.	At4g37280	CGC CTA CCA TGG TCC TCG C	CTT CCA CTC CTT CTT ACG AAG CTC
133.	At4g39350	ACT GGT GGT CGA CTC ATT GC	TTT GCA CTG AGG ACA AGC TTG
134.	At5g01220	TTGGCGTAGAAAAGAGTTTGGAGCT	GTCGTTGCTGCTCTCCAATCATA
135.	At5g02120	CTTTCGACCTTGTCGGCTCAT	ATGAGACCAATCATGCAAGCTC
136.	At5g03770	CTCAAATGCGACAGAGCACC	ATGATGTGGATGTCGAGGCAC
137.	At5g04920	TGGAACGGTGAGGATGGTGG	CCAGCTTGTTCTAATGGGATTC
138.	At5g08280	CGAACTGCTATCATCAGAATTGG	CCGTTTATCAAGGCCTCGTC
139.	At5g09310	GAGGAAGCATCGGTGGATTAC	CGAAAGAAGAGCTGTAAAGACACT
140.	At5g10070	TGTGATGCAAAGAGGTGCAC	GCAGATAAAGCCTCCACACA
141.	At5g13030	TAACCTGGGACCACTCCTTTGTT	GACCATGCTACAAGCTGAGGATCA
142.	At5g13480	GATCTTGCATGGCATCCTATTGG	CTCCAGGAGTTGGCGATGC
143.	At5g15400	TGGTTAGTCATGAGTGGTGGGTT	GGACGACGTTCCGATGCT
144.	At5g15750	GATCCTTTTCGTATTCAGATGACTG	CACGAATGTGTCCTTGCTCTATG
145.	At5g17410	GACCAATTTGTGGAGTCAAGCTC	ATGTTCCAGTTGTGCCACCAT
146.	At5g17840	CGTGCTCTCTTCCTCCTCCT	CCGTTTAAACATGACTCAGCCTA
147.	At5g18200	TCTTAGCAGGAATCTCGAGACC	TGAAGCATGAGTTTCAGAAGTCC
148.	At5g22640	ATTGAGGAGTTTCTTCAGTGGGT	GACATCCATTTCCAACCACTT
149.	At5g24314	CAGAGAGATGATAATGGACGCC	CCACAAACGGAACTCTTTGC
150.	At5g24490	TCGTGGGACGGTCCTCTTG	CACCATGCTTCTTTGTAAACAATGT
151.	At5g25080	GGCTAAACGTGTACAGAGAGAAG	GCTGCCTGACGGTTTAAGAC
152.	At5g27380	GCTCGTCGTTGGTGACAAAA	ACCGGTGTAAACCCAAACGAA
153.	At5g27830	CCTGTTTCTTCATCTCCTGATCTC	GCTTCTAGACTTGCTTTGCTTCC
154.	At5g28750	GTGATTGCTGGCGTCG	GTCTTAAGCTCAGACTCAAACTCTT
155.	At5g47890	GATCCAGCCTCAGATGTGG	TCCAAGTTTACACACCTCTCCAC
156.	At5g49510	GAAGCAAGGAAGGGTACTGG	CCAACCACAAACACACTGAGTC
157.	At5g54750	TTCACGTTGACGTATGGTGC	CCAGCTTGACACTGATGCAG
158.	At5g58730	CATTGTTAGGGAACCTCTTTGG	CTGCTTCTTCCTTCTCTGTACCTC
159.	At5g65220	CAGAGCGAAGACGACGGAG	GTCAAGTTTCCTCGACAACCT
160.	At5g66290	TTGAGCTCTTCTCCATTTACCG	GCAGTTGTTGATCACCTCTGTC
