# Supplementary material for: Genetics and Molecular Mapping of Black Rot Resistance Locus Xca1bc on Chromosome B-7 in Ethiopian Mustard (Brassica carinata A. Braun)
Source: PLoS One. 2016 Mar 29;11(3):e0152290. doi: 10.1371/journal.pone.0152290 (PMC4811439; doi:10.1371/journal.pone.0152290)
Supplement: S4 Table — (PDF) [file pone.0152290.s004.PDF]

S4 Table: List of polymorphic markers

S.N.	Marker Id	Forward Sequence (5'-3')	Reverse Sequence (5'-3')
1.	Na10-D07	CTACTTTGATGGACACTTGCC	TCTGAAGTTGATTAGTCGGTCC
2.	Na14-G02	TTCCCTTTATTGAGCAAGCTG	TCCCGGTCGCTAAGATATTG
3.	BRAS61	GCAGCCTTCAACTCCCATAGA	TGGGTTCGAGCAGGGTTC
4.	Ni3-C08	CCCTAACACGGTGTCAACAG	GGCAGAATCATCGAGAGGTC
5.	BRAS116	TTTCCACCACCACCGCCATT	GGAAGCAGATGGAGACGGAG
6.	Ni2-D10	GATGCCCCAAATCTGTTACG	CAATTCGTGAAAAATAGCCG
7.	BnGMS091	ACGCATTCTCTGAATTGACT	GAGCAGTGAGAAAGTCTTCG
8.	BnGMS440	TCAATGTTTATTCCACCCTC	CTTCTTCCGACAAGTCTTTG
9.	Ni2-D08	TTTAGGGAAAGCGAATCTGG	ACAACAACCCATGTCTTCCG
10.	Na14-A06	GCCTGTCTTCTCCTCCACAC	ACATTGGATTATGCCCGTTG
11.	Ni4-C10	AGATGCTAAAGCGGATCACC	CACTTGGTAACTCTATGGATGCC
12.	Na10-D03	ATGATTTGCCTTGAAATGCC	GATGAAACAATAACCTGAGACACAC
13.	BnGMS298	GGTGCATACTACTTAAGCCC	AGCATGGTACGATTCAGTTT
14.	BnGMS679	ACAGAGAGAATGAGAATGCG	GAAGAAGGACGCAATCATAG
15.	Ni3-A05	ATCGGACAGACTCCTCTTGC	TACCCCCTCTGCATCTTAGG
16.	Ni2-C01	GAGTATGAGAGATGGGAATCCG	GACTGAGCAGCTTGGAGACC
17.	Ni2-A02	GAGTGGAATCTTGCTACTGTCG	AAGGTCTGTGGAAATGACAGG
18.	CB10439	ACCTCGAAGGGTATCTGC	CGTGCAATTTCAACAACA
19.	Ni2-C09	ACGGAAGAAATCCAACCTCG	TATGCTTGGAAATGGTTTGG
20.	BnGMS277- A	GTAGAGGATGATAATTGCGG	ACACGTGCTATCTCGTCTCT
21.	Na10-H03	GAGCTGGCTCATTCAACTCC	CACAATTTCTCAGACAAAACGG
22.	Ni4-B04	TAGGCGGACTTGTTGATTCC	CATGATTCAGAACAAGGGTGC
23.	Ol11-D12	CCTCCACCGCACTCAATTAC	TGGAGAAGTTTGGGACATTTTC
24.	Ni4-B03	ACTTTCTTTACATTCTAATCGC	GCTGTTTCTGTGGAATTGTCG
25.	BnGMS277- B	GTAGAGGATGATAATTGCGG	ACACGTGCTATCTCGTCTCT
26.	MR153	AAC AGA AGA CAT GCG GAA AC	ATT GAT TTG AGC CGG TAA AGA
27.	Ni4-A05	AAGGGGTTTGTTGTTGTTGG	GGCGCATTAGATTGTCTGG
28.	Ni2-A07	GGAACCCAACAAGTGAGTCC	AGAGCTTGAGACACATAACACC
29.	Ol10-D08	TCCGAACACTCTAAGTTAGCTCC	GAGCTGTATGTCTCCCGTGC
30.	BnGMS289	CATTACAAACTCAGCGTCAA	CAGGACACTCGGTTATCAAA
31.	Ni2-D03	CGTATGTGAAAAATAAATGG	TTGAGCTTGAGATCATCCCC
32.	At3g63420	AAGCACATGATCCTTGCGGAGC	CTTCAAACCACCGGTCCCATCC
33.	At1g47570	CGGAAAGACATGAATGGTGCAG	CTTGAATCTGGAGCAGGCATTA
34.	At3g55005a	GAGATGATGGATCTCAAGACC	GAACTTCAAGAATCCTTCAAGA
35.	At4g33140	CCA TTC AGT CTC AGA ATA CCA TGT C	TGT CTT AAA GAA TTC ATG AAC ACG T
36.	At1g70610	TGGGTTATCTTCGCTGCGTT	GTCACCAACAGTTTGAGAGTCGA
37.	At1g71865	TTGCGTCTCCAGATCTCAA	GATCTTGCAGCTTGAATGAGTGA
38.	At1g72380	TGCAGATCATGAAGTTTCCTT	ACGTTTCCGATAGTCATGCA
39.	At5g65220	CAGAGCGAAGACGACGGAG	GTCAAGTTTCCTCGACAACCT
40.	At5g66290	TTGAGCTCTTCTCCATTTACCG	GCAGTTGTTGATCACCTCTGTC
41.	At5g25080	GGCTAAACGTGTACAGAGAGAAG	GCTGCCTGACGGTTTAAGAC
